# Seasonal Variation in Root Morphological Traits and Non-Structural Carbohydrates of *Pinus yunnanensis* Seedlings Across Different Seedling Orders

**DOI:** 10.3390/plants14050825

**Published:** 2025-03-06

**Authors:** Zixing Pan, Zhuangyue Lu, Sunling Li, Jianzhen Liao, Chiyu Zhou, Lin Chen, Shi Chen, Nianhui Cai, Dexin Wang, Yulan Xu

**Affiliations:** 1 Key Laboratory of National Forestry and Grassland Administration on Biodiversity Conservation in Southwest China, State Forestry Administration, Southwest Forestry University, Kunming 650224, China; pzixingg@163.com (Z.P.); luzhuangyue@swfu.edu.cn (Z.L.); 18313173051@163.com (J.L.); zhouchiyu@swfu.edu.cn (C.Z.); linchen@swfu.edu.cn (L.C.); chenshi@swfu.edu.cn (S.C.); cainianhui@swfu.edu.cn (N.C.); 2 Key Laboratory of Forest Resources Conservation and Utilization in the Southwest Mountains of China, Ministry of Education, Kunming 650224, China; 3 Yunnan Academy of Forestry and Grassland, Kunming 650224, China; lisunling@yafg.ac.cn

**Keywords:** non-structural carbohydrate, seasonal variation, root morphological traits, *Pinus yunnanensis*, seedling orders

## Abstract

Non-structural carbohydrates (NSCs), comprising soluble sugars (SS) and starch (ST), are essential for plant growth and development. The distribution of SS and ST concentration across various organs fluctuates throughout time due to the changes in root morphology in plants, ultimately demonstrating multiple strategies for adapting to seasonal environmental variations. The purpose of this investigation was to explore the seasonal dynamic patterns of root morphology in *Pinus yunnanensis*, with particular emphasis on specific root length (SRL), specific root surface area (SRA), root tissue density (RTD), and average diameter (AD). This study also aimed to investigate the seasonal fluctuation patterns of NSC. The SRL, SRA, RTD, and AD in both first-order and second-order seedlings had analogous fluctuation patterns from March to December. Although the SRL, SRA, RTD, and AD of third-order seedlings exhibited minor differences from the preceding orders, the overall variance patterns corresponded with those of the first two seedling groups. Consequently, the seasonal fluctuations in SS, ST, and NSC levels in various seedling orders exhibited patterns similar to root morphological characteristics. The SRL, SRA, and AD of three seedling orders exhibited a significant correlation with SS, ST, and NSC, confirming the link between NSC concentration and root morphology. The responses of SS, ST, and NSC in various organs of *P. yunnanensis* seedlings to root morphological characteristics further substantiated the correlation between the variations in NSC across different organs and root morphological traits.

## 1. Introduction

Biomass allocation to different organs could contribute to plant growth [1,2]. The allocation of biomass between aboveground and belowground components significantly influences several physiological processes in individual plants [3,4,5,6]. According to the optimal partitioning theory [7,8,9], plant organs that absorb the most limiting resources are likely to obtain a comparatively bigger allotment of biomass [10,11,12]. In woody plants, differential growth rates resulted in a discrepancy in wood accumulation, causing a progressive rise in aboveground biomass relative to belowground biomass [5]. These observations validated the trade-offs between aboveground and belowground biomass.

The concentrations and accumulations of non-structural carbohydrates (NSCs) differed among organs, resulting in differences in biomass within those organs. Root development may fluctuate on a monthly basis [13], and plants may rapidly mobilize and allocate NSC (e.g., starch and soluble sugars) stored in roots to aerial organs [14]. The allocation of NSC affects biomass distribution, indicating the specific growth circumstances of plant organs.

NSC is a crucial component participating in physiological processes. NSC comprises soluble sugars (SS) and starch (ST) [15], functioning as markers of the carbon supply status in plants, which ultimately influences their development [16]. In perennial woody species, despite variations in the quantities and collections of NSC across stems and roots, these organs serve as the primary reservoirs of NSC in plants [17]. These preservatives function as essential compounds that support the growth of woody plants [16,18]. In recent decades, researchers have extensively investigated the correlation between NSC and root absorption ability. Researchers anticipate that global temperature change will influence carbon trade-offs and physiological functions in plants [19,20]. Consequently, several researchers have focused on elucidating the impact of NSC on root absorption ability during drought stress. To endure drought stress, plants modify SS concentration to regulate physiological processes [21,22]. Yang et al. [23] showed that thick roots have increased NSC accumulation under drought circumstances. Blumstein et al. [24], utilizing a database of NSC measurements, noted that while environmental stress influenced the total NSC stored by a plant, the capacity of its roots to absorb carbon may exert a more significant impact.

Root traits can modify the absorption and transport efficiency of water and nutrients, significantly influencing plant growth and survival strategies [25,26,27]. Specific root area (SRA, root surface area per mass), specific root length (SRL, root length per mass), and root tissue density (RTD, mass per root volume) are critical root morphological characteristics that influence resource absorption rates, root respiration, and rhizodeposition [28,29,30,31]. Weigelt et al. [32] discovered that an increased RTD resulted in greater tissue investment. Weemstra et al. [33] showed that the SRL and SRA typically indicated the root uptake area at a certain biomass cost and nutrient absorption capability. Roots can modify their morphological, chemical, and physiological characteristics, which are functional traits, to respond to environmental changes [34]. Plants often enhance resource acquisition by extending total root length (RL) to optimize soil use and efficiently exploit available resource patches [35,36] or by modifying root morphological traits (e.g., higher SRL) to enhance nutrient absorption efficiency [36,37,38,39].

Previous studies indicate that increased carbon uptake correlates with an enhancement in root surface area and root length [40,41]. NSC plays a crucial role in regulating plant physiological processes across various environmental conditions. Water [42,43,44,45], CO_2_ content [46], and shade [45,47] are the primary factors considered in most studies when assessing NSC dynamics at the tissue or organ level. In addition to aboveground reserves, sufficient belowground reserves in woody plants contribute to their resistance and resprouting capacity [48]. Resprouting is a prevalent strategy among conifers [49] and is considered a derived state [50]. Starch reserves in woody underground organs, particularly roots, are essential for re-sprouting [51,52]. Subsequently, additional studies began to examine the correlation between root traits and NSC. The results for *Fraxinus mandshurica* indicate that elevated root length is facilitated by reduced tissue density [53]. Additionally, the observed negative correlation between root tissue density and root length is a prevalent trend across various tree species [54]. Mei et al. [55] conducted a study that determined the biomass and seasonal dynamics of NSC in leaves, stems, and roots. The results indicated that non-absorptive thick roots mobilized stored NSC to support absorptive thin roots. Additionally, species with smaller belowground NSC reserves exhibited a more significant decline in root NSC storage following stem girdling. Recent studies have concentrated on root traits, particularly the correlation between root morphology and NSC content in woody plants [22,56], which have demonstrated that NSC dynamics are linked to belowground reserves. These studies have examined the relationship between woody plant root morphology and NSC reserves in influencing plant resistance capacity to drought stress. The results of current and previous studies indicate the potential to investigate the relationship between root morphology and NSC storage in relation to plant survivability.

*Pinus yunnanensis* is one of the abundant tree species in southwest China, accounting for approximately 52% [57] of the forest area in Yunnan Province. *P. yunnanensis* is a species within the forest community that prefers high light conditions. Its tolerance for barren areas and low temperatures provides substantial ecological, economic, social, and research benefits to the region [58]. Seedling order is an indicator to evaluate the quality of *P. yunnanensis* seedlings based on plant height. *P. yunnanensis* seedlings with a higher plant height would be classified as high quality and graded into a lower order. Accordingly, the *P. yunnanensis* seedlings were arbitrarily divided into three height-based orders. During the growth stage, the mortality rate of *P. yunnanensis* seedlings rises exponentially as rainfall decreases, due to the underdeveloped root system’s sensitivity to surface soil moisture [59]. Consequently, studying the role of NSC content in influencing root uptake in *P. yunnanensis* seedlings is essential. A number of investigations have examined the absorption issues associated with the underdeveloped root systems of *P. yunnanensis* seedlings, focusing on morphological and growth changes as well as metabolic–physiological regulation [60,61]. Additional studies have validated that NSC serves as a connection among allocation, metabolism, and uptake stresses [62,63]. Numerous studies have investigated the effects of nutrient concentrations and stoichiometric characterization in *P. yunnanensis* [64,65,66,67,68,69]. Recent research has shifted to examining the relationship between NSC content and the stoichiometry of carbon, nitrogen, and phosphorus, along with the implications of NSC storage and root morphology [22,70]. To our knowledge, limited research has examined the response of NSC concentration in *P. yunnanensis* to variations in root morphological traits across different grading orders.

This study aimed to investigate the influence of root morphological traits across various seedling orders on the variation patterns of NSC concentration, ultimately affecting the seasonal growth dynamics of *P. yunnanensis*. The hypotheses proposed were as follows: (1) seedlings with thinner roots will modify their root morphological traits to enhance water and nutrient absorption; (2) roots from different seedling orders will utilize SS and primarily store ST to improve water uptake during winter drought conditions; and (3) variations in NSC across different organs may be closely associated with root morphological characteristics.

## 2. Materials and Methods

### 2.1. Description of the Experimental Zone

The experiment was conducted at the greenhouse of Southwest Forestry University in the Panlong District of Kunming, Yunnan Province (25°04′00″ N, 102°45′41″ E). The experimental site, situated at an altitude of approximately 1945 m, experiences a subtropical semi-humid plateau monsoon climate. The mean annual temperature is 14.7 °C, with an absolute maximum of 32.5 °C and an absolute minimum of −9 °C. The average annual relative humidity is 68.2%, with annual precipitation ranging from 700 to 1100 mm. Precipitation is primarily concentrated from May to October, featuring a maximum monthly rainfall of 208.3 mm and a maximum daily rainfall of 153.3 mm. The average annual sunshine duration is 2445.6 h, with a sunshine rate of 56%. The experimental site exhibits a significant solar projection angle year-round, with an average annual total radiation of 543.36 kJ/cm^2^ and an average annual evaporation of 1856.4 mm.

### 2.2. Source of Sapling Materials

The sapling seeds were collected from a *P. yunnanensis* asexual seed orchard in Maidu County, Yunnan Province, and the mother tree was in good growth condition. The seeds with full and uniform grains were selected. Seeds were uniformly sterilized with a 0.5% potassium permanganate solution for 1 h, removed and rinsed with water, and then soaked in warm water at 50 °C for 24 h. Similarly, the soil and seedling trays were sterilized with potassium permanganate before sowing, and seeds were planted at the end of January 2022, with 2–3 seeds in each hole. After sowing, the pine needles were spread on seedling trays, and the arch was used to build the greenhouse skeleton, which was then covered with a film to fix it. Greenhouse management included the regular implementation of tasks such as watering, weeding, and other necessary measures in accordance with prevailing weather conditions and the developmental stages of the seedlings. After the seeds had fully sprouted, transplant the seedlings into nursery pots (container size 18 cm base diameter × 32 cm height) in May 2022, with a soil substrate ratio of humus/laterite = 2:1. Individual seedlings of uniform size were selected for the late stage of the experiment, during which the seedlings were managed uniformly, weeding every two weeks, watering every 3–5 days, and ensuring that the soil was watered thoroughly each time.

### 2.3. Field Deployment and Seedling Grading

All seedlings were cultivated in the pots under uniform environmental circumstances in the nursery garden of the greenhouse at Southwest Forestry University, and over 1900 seedlings were assessed for grading post-cultivation in March 2023. The grading criteria were determined by the development status of seedlings, utilizing the mean ± 1/2 standard deviation approach as the average growth type, which was classified into three categories (1st-order seedlings, 2nd-order seedlings, and 3rd-order seedlings). The calculation formula is as follows:

θ = ω ± 1/2σ,(1)

where θ is the order of *P. yunnanensis* seedlings, ω is the average height of the seedlings, and σ is the standard deviation of the population of seedling heights.

**Figure 1:**
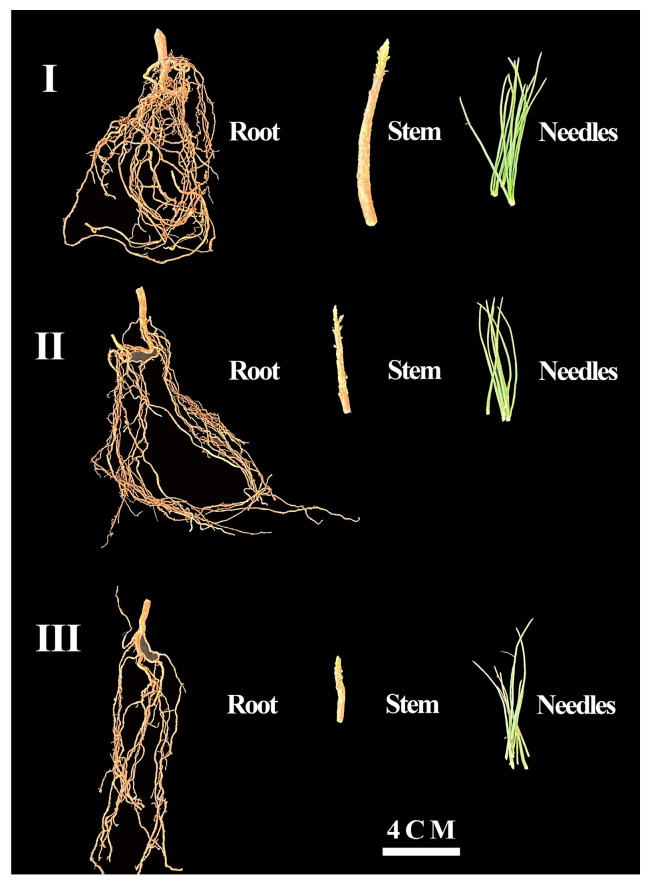
The *Pinus yunnanensis* seedlings of different orders. I: The first-order seedlings; II: the second-order seedlings; and III: the third-order seedlings.

Seedling order measures the quality of seedling growth based on plant height. National standards (LY/T 1950-2011) for the development of seedling quality indicators as the basis for grading [71], the range of sizes of the 1st-order seedlings (Figure 1) was calculated by using the standard formula for the division as follows:

I > ω + 1/2 σ,(2)

The 2nd-order seedlings (Figure 1) were calculated as follows:

ω − 1/2 σ ≤ II < ω + 1/2 σ,(3)

And 3rd-order seedlings (Figure 1) were calculated as follows:

III < ω − 1/2 σ(4)

After grading, all the different orders of seedlings were used for planting incubated under uniform circumstances for further experiments.

### 2.4. Analysis and Measurement

#### 2.4.1. Growth Measurement

Measurement of data related to basic indicators (seedling height and stem diameter at ground level) began in March 2023 (when the seedlings were 420 days old), and up to December 2023 (when the seedlings were 690 days old), measurements were taken once a month for a total of 10 measurements. Seedling height was measured by straightedges (accuracy 0.1 cm), while stem diameter at ground level was measured by electronic vernier calipers (accuracy 0.01 mm) to determine phenotypic data for one growth cycle of the seedlings. The growth changes in seedling height, stem diameter at ground level, and other relevant indexes of *P. yunnanensis* seedlings were analyzed using SPSS Statistics 26 analysis software of IBM to evaluate the differences in growth patterns among seedlings of different orders.

#### 2.4.2. Individual Biomass Measurement

Seedlings were assessed to ascertain individual biomass, and a fully randomized block design was applied to collect experimental samples. Three seedlings were selected as biomass-measuring specimens, with three biological replicates in each of three blocks for each order, resulting in a total of 27 seedlings throughout the three orders per timepoint. These seedlings were measured once a month during March (420 days), June (510 days), September (600 days), and December (690 days) of 2023, for a total of four measurements. The experiments were conducted using the whole plant sampling method (without destroying the seedling tissue) for digging the seedlings, carefully rinsing the root soil with water to keep the root system intact, and then bringing them back to the laboratory to air-dry in a cool place. The sampling plants were divided into three parts: roots, stems, and needles. The fresh weight of each component was weighed and recorded. The measured samples were placed in paper bags and labeled, after which they were put into an oven at 105 °C for 30 min, and then the temperature was adjusted to 80 °C for drying treatment until the mass was constant. The dry weight of each component, which was the biomass of each component, was weighed with an electronic balance with an accuracy of 0.001 g, and the data were recorded.

The aboveground biomass was calculated as follows:

(5) Aboveground biomass=stem biomass+needles biomass

And the individual biomass was calculated as follows:

(6) Individual biomass=root biomass+stem biomass+needles biomass

The biomass allocation ratio refers to the ratio of the biomass of a component to the total biomass of a single plant. A differential analysis of *P. yunnanensis* biomass was carried out to observe the differences between biomass allocation of different orders of *P. yunnanensis* seedlings.

#### 2.4.3. Soil Chemical Characteristics Measurement

In September 2022 (240 days), three uniformly grown seedlings with three biological replicates were randomly selected from each order to obtain soil samples. Soil was extracted from each of the nine seedling pots of identical order. The collected soil was thoroughly blended and utilized as a sample for that order. Soil samples were dried and ground through a sieve. For subsequent determinations, 0.2 g of each soil sample from each order was used. The H_2_SO_4_-H_2_O_2_ decoction method was used to decoct the collected samples to obtain the solution to be measured. N and P contents were determined using an auto discrete analyzer provided by Shenzhen E-Zheng Tech Co., Ltd. (Shenzhen, China). C content was determined by the potassium dichromate titration method, and K content was determined by the flame photometric method [72].

#### 2.4.4. Root System Measurement

A fully randomized block design was applied to collect experimental samples. Three seedlings were selected as biomass-measuring specimens, with three biological replicates in each of three blocks for each order, resulting in a total of 27 seedlings throughout the three orders per timepoint. After digging out the seedlings for the biomass determinations as described above, and before proceeding to dry, the root systems were scanned with an LA-S root scanner. After scanning, the root images were analyzed with LA-S plant image analysis software (ver. 2000) provided by Wanshen Detection Technology Co., Ltd., Hangzhou, China. The data were derived from a table of root morphology indexes for the total root length, root surface area, average root diameter, and root volumes, respectively.

#### 2.4.5. Measurement of Soluble Sugar and Starch Content

Saccharides represent a fundamental component of plant bodies and function as the primary storage substrate for metabolic processes. The anthrone colorimetric method was used for the measurement, and the detection kit was provided by Michy Biomedical Technology Co., Ltd., Suzhou, China (M1503A). The experimental steps were carried out according to the experimental instructions of the detection kit. *P. yunnanensis* seedlings with good growth conditions under each treatment were randomly selected at 420 days, 510 days, 600 days, and 690 days, and their dry samples were used for the measurement. Three seedlings with three biological replicates for each order were dug out as measuring plants, resulting in a total of 27 seedlings for the three orders. The method of Buysse and Merckx [73] was referred to for determination and calculation.

Starch is a nutrient stored in the plant body, and determining its content is important for investigating sugar metabolism in plants. The content of starch was determined by a detection kit (M1101A, Michy Biomedical Technology Co., Ltd., Suzhou, China), which is based on anthrone–sulfuric acid colorimetry. The experimental steps were carried out according to the experimental instructions. *P. yunnanensis* seedlings with good growth conditions under each treatment were randomly selected from 420 days to 690 days, and their dry samples were used for measurement. Three seedlings with three biological replicates for each order were dug out as measuring plants, resulting in a total of 27 seedlings for the three orders. The method of Xie et al. [74] was referred to for determination and calculation.

The calculated soluble sugar content and starch concentration were used to calculate the total NSC concentration according to the following formula:

NSC Concentration=soluble sugar concentration+starch concentration.

#### 2.4.6. Measurements of Root Morphological Traits

The phenotypic plasticity index (PI) for each root morphological trait was determined using the approach outlined by Valladares et al. [75] with the subsequent formula:

(7) PI=[max(i)-min(i)]/max(i)

Max(i) denotes the maximum value of the functional trait under a treatment, whereas min(i) signifies the minimum value of the functional trait under a treatment.

The coefficient of variation (CV) for root morphological traits was calculated according to Zhang et al. [76] using the following formula:

(8) CV=SD/mean × 100%

(9) Specific root length (SRL)=RL/RDW

(10) Specific root surface area (SRA)=RA/RDW

(11) Root tissue density (RTD)=RDW/RV

RL denotes root length, RA signifies root surface area, RV indicates root volume, and RDW refers to the mass of the root system desiccated to a constant weight and subsequently measured dry.

#### 2.4.7. Statistical Analysis

SPSS Statistics 26 analysis software of IBM was used to analyze the statistics of this study. The statistical methods used were two-way ANOVA, redundancy analysis (RDA), and Pearson correlation analysis. Duncan’s multiple comparison method was used for significance tests (α = 0.05).

## 3. Results

### 3.1. Soil Chemical Characteristics of Different Seedling Orders

The C, N, P, and K contents in soil samples from different orders showed similar absorption patterns (Table 1). The K contents among three seedling orders were higher than C, N, and P contents. The C contents were the lowest compared to other nutrient elements. The N contents and P contents of the same seedling order were generally at a consistent level.

The C and P contents in the soil from different orders exhibited analogous absorption trends. The concentrations of C and P in the first-order soil samples were the highest. The N and K contents of the first-order soil samples showed lower trends compared to other orders.

### 3.2. Allocation of Biomass to Different Organs Among Different Seedling Orders

**Figure 2:**
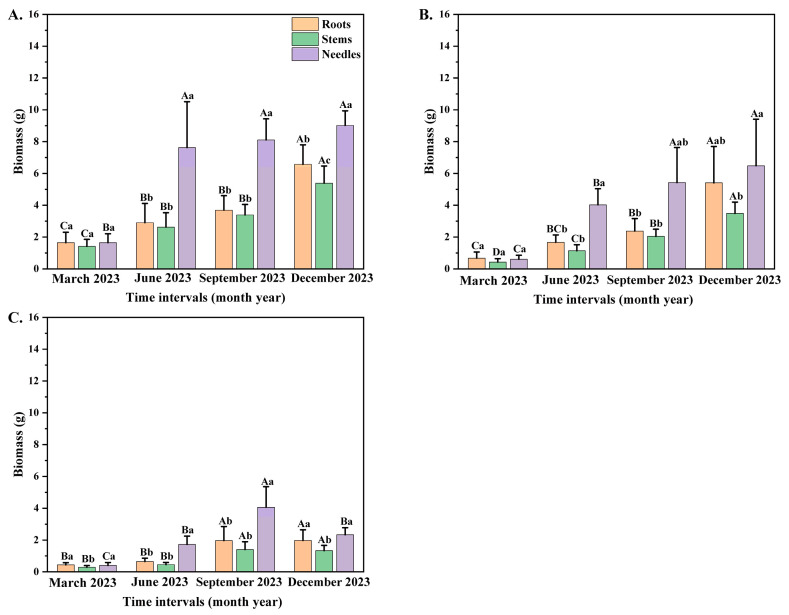
The biomass allocation of different *P. yunnanensis* seedling orders among different months. (**A**) First-order seedlings; (**B**) second-order seedlings; and (**C**) third-order seedlings. The different uppercase letters indicate significant differences among time intervals (*p* < 0.05). The different lowercase letters indicate significant differences among different organs (*p* < 0.05).

Time interval and seedling orders significantly correlated with the individual biomass of *P. yunnanensi seedlings* (Table 2). With the increase in seedling orders, the individual biomass of *P. yunnanensis* seedlings showed progressive decreases during different sampling dates. In contrast, the biomass of different organs generally showed significant increasing trends under the same seedling order (Figure 2A–C). During the same month, the biomass with all seedling orders was the highest in needles and the lowest in stems. On the other hand, this difference in biomass allocation between stems and needles is meaningful, and the relationships of biomass allocations over time are important data. The biomass in needles of the first two seedling orders increased significantly in June (Figure 2A,B), whereas the third-order seedlings increased markedly in September (Figure 2C). The stem and root biomass in the first two orders increased greatly in December, while third-order seedlings increased significantly in September. The present study examined the biomass of the first-order, second-order, and third-order seedlings in various organs, with a focus on the timing of peak biomass accumulation. The results indicated that the biomass of the first-order, second-order, and third-order seedlings exhibited a progressive increase, reaching its peak in December. The analysis further revealed that, among the seedlings of the three distinct orders, the biomass allocated to needles was the most substantial, followed by roots (Figure 2A–C).

The biomass of second-order seedlings had the most significant coefficient of variation among distinct periods of time, with values of 89.63, 39.06, and 35.67 in roots, stems, and needles. With the growth in seedling orders, the coefficient of variation of the second-order seedling biomass among different months was the most significant, with values of 53% (in March), 63% (in June), 62% (in September), and 48% (in December) (Figure 2B).

### 3.3. Root Morphological Traits Observed Among Different Seedling Orders

**Figure 3:**
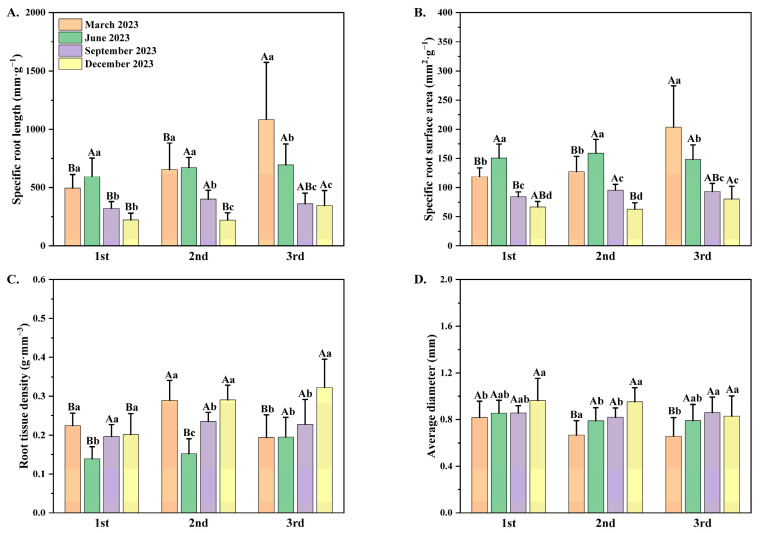
(**A**) The specific root length (SRL); (**B**) specific root surface area (SRA); (**C**) root tissue density (RTD); and (**D**) the average diameter (**A**,**D**) of *P. yunnanensis* seedlings in different seedling orders. The different uppercase letters indicate significant differences among seedling orders (*p* < 0.05). The different lowercase letters indicate significant differences among time intervals (*p* < 0.05).

Time intervals and seedling orders have a significant correlation with the specific root length (SRL), specific root surface area (SRA), root tissue density (RTD), and average diameter (AD) of *P. yunnanensis* seedlings (Table 2). As orders for *P. yunnanensis* seedlings rise, both SRL and SRA exhibited significant upward trends at the same timepoint (Figure 3A,B). As dates of measurement progressed, both SRL and SRA had notable declining tendencies. Overall, the RTD of three different orders demonstrated initial increases followed by subsequent declines as dates of measurement progressed. The AD displayed general upward trends as dates of measurement progressed (Figure 3C,D).

**Figure 4:**
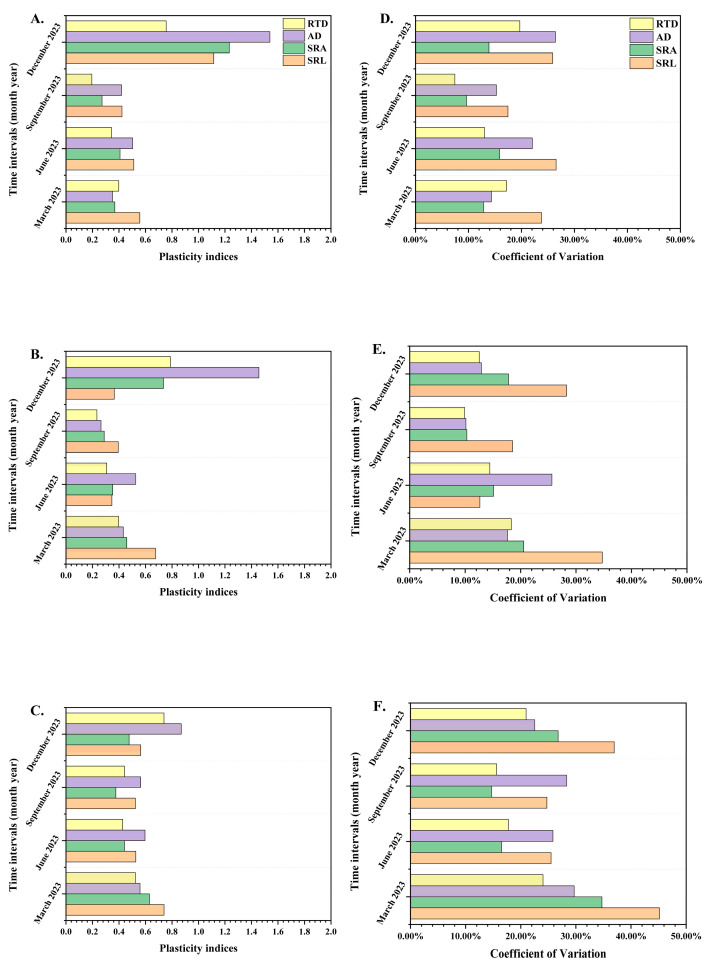
The plasticity indices of different *P. yunnanensis* seedling orders among different months (**A**–**C**) and the coefficient of variation of different seedling orders among different months (**D**–**F**). First-order seedlings (**A**,**D**); second-order seedlings (**B**,**E**); and third-order seedlings (**C**,**F**). SRL: specific root length; SRA: specific root surface area; RTD: root tissue density; AD: average diameter.

The plasticity indices (PI) for RTD, AD, SRA, and SRL indicated that, as seedling orders increased, the PI generally exhibited a decreasing trend (Figure 4A–C). The average values for PI across different orders were 0.587, 0.501, and 0.562, respectively. Among the three seedling orders, the PI of AD had the highest value.

With the rise in seedling orders, the coefficient of variation (CV) showed increasing trends (Figure 4B,D,F). The average values of CV from the first-order to the third-order seedlings were 18%, 17%, and 26%, and the average values of CV in the first-order and the second-order were lower than 20%, which demonstrated the first-order and the second-order seedlings showed internal homeostasis in root morphological traits [23,76].

Under the identical period, the CV of SRL, SRA, RTD, and AD in the third-order seedlings were the highest, which were 45%, 35%, 30%, and 24%. Among the three seedling orders, the CV of SRL had the highest values, which were 27%, 35%, and 45%.

### 3.4. Seasonal Variation of Root Morphological Traits Among Different Seedling Orders

The SRL and SRA of the first-order and the second-order seedlings had the same seasonal variation patterns (Figure 3A,B). The SRL and SRA in the first two seedling orders increased from March to a maximum in June and then decreased to a minimum in December, while the SRL and the SRA in the third-order seedlings showed increasing tendencies and peaked in December. The RTD of all seedling orders decreased from March to a minimum in June and then showed increasing trends (Figure 3C), whereas the AD of three seedling orders increased from March until peaking in September or December (Figure 3D). The seasonal variation patterns in SRL and SRA of *P. yunnanensis* seedlings among different orders were similar: first-order and second-order seedlings had the same seasonal variation trends, whereas the seasonal dynamic of third-order seedlings differed from those of the first two orders of seedlings. The first two orders of seedlings showed an increasing and then a decreasing trend. Third-order seedlings exhibited a decreasing tendency from March to December. Seasonal variation in RTD and AD of *P. yunnanensis* seedlings also showed consistency: seedlings of all three orders displayed the same dynamic patterns among different months (Figure 3C,D).

### 3.5. Allocation of NSC Concentration to Different Organs of Seedlings Among Three Orders

**Figure 5:**
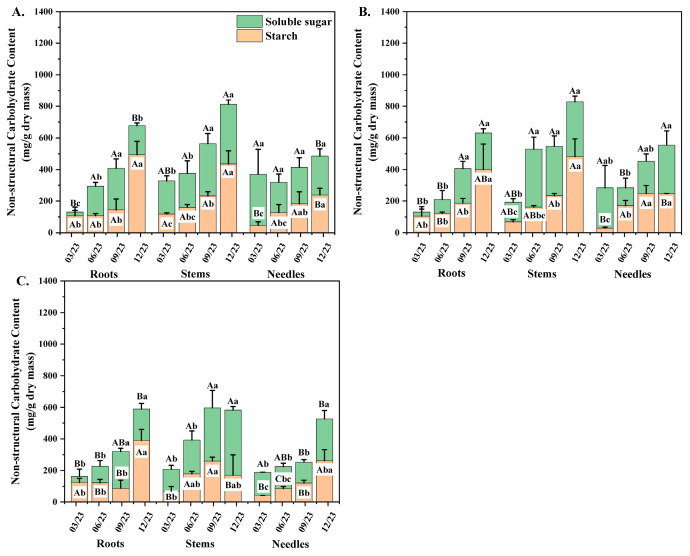
The NSC concentration of different *P. yunnanensis* seedling orders in organs among different sampling dates. (**A**) First-order seedlings; (**B**) second-order seedlings; (**C**) and third-order seedlings. The different uppercase letters indicate significant differences among different organs (*p* < 0.05). The different lowercase letters indicate significant differences among time intervals (*p* < 0.05).

Time interval and seedling orders have significant correlation with soluble sugar (SS), starch (ST), and NSC of *P. yunnanensis* seedlings (Table 3). With the increasing seedling orders, the SS and ST concentrations per unit dry mass in roots and stems, respectively, showed decreasing tendencies in general (Figure 5A,B). Within the same seedling order, SS concentration in roots and stems of seedlings was generally higher than that of needles. With ascending seedling orders, the ST concentration of first-order and second-order seedlings in roots and stems were 30%, 37%, 15%, and 27% greater than those of needles, respectively (Figure 5A–C). The total NSC concentration per unit dry mass of all three seedling orders in stems was 38%, 82%, 52%, 75%, 37%, and 49% higher than those of roots and needles, respectively. The ST and total NSC concentration of lower-order seedlings between stems and roots varied considerably (Figure 5A,B). Third-order seedlings exhibited the highest SS-ST ratio in roots compared with the first two seedling orders, and the SS-ST ratio in stems showed progressively increased trends with increased seedling orders (Table 4).

### 3.6. Seasonal Variation of NSC Concentration in Different Organs Among Different Seedling Orders

In the needles of *P. yunnanensis*, the SS concentration of second-order and third-order seedlings peaked in December, whereas the ST concentration showed increasing trends until peaking in September or December (Figure 5A–C). In stems, the SS concentration of three seedling orders increased from March to a maximum in December, and the ST concentration of the first two orders showed the same increasing patterns. In contrast, third-order seedlings increased from March to a maximum in September and then decreased (Figure 5A–C). The total NSC concentrations of aboveground organs showed similar seasonal variation in the first two seedling orders. The NSC concentration in the needles of first-order and second-order seedlings decreased from March to a minimum in June and then increased until peaking in December, while third-order seedlings showed an increasing trend and peaked in December. The NSC concentration in stems of first-order and second-order seedlings showed increasing trends and peaked in December, whereas third-order seedlings increased from March to a maximum in September and then decreased. Unlike the aboveground organs, the root NSC concentrations of three seedling orders showed increasing trends during different time intervals (Figure 5A–C). The SS concentration of all seedling orders increased in March and peaked in December. The minimum ST concentrations of third-order seedlings occurred in September, and the maximum was found in December, while the NSC concentrations of the first two orders increased from March and peaked in December.

### 3.7. Relationship Among the Individual Biomass, Root Morphology, and NSC Concentration of Three Seedling Orders

**Figure 6:**
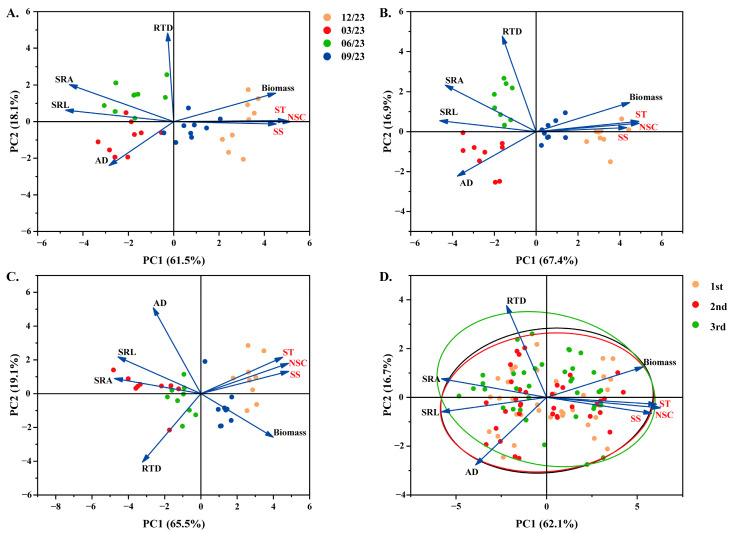
Redundancy analysis of root morphology traits and NSC in different *P. yunnanensis* seedling orders among different time intervals. (**A**) First-order seedlings; (**B**) second-order seedlings; (**C**) and third-order seedlings; (**D**) Redundancy analysis of three seedling orders during the whole experiment period. SRL: specific root length and the ellipses in this figure represent the area of distribution of the samples of different orders of seedlings; SRA: specific root surface area; RTD: root tissue density; AD: average diameter; SS: soluble sugar; ST: starch; NSC: non-structural carbohydrates.

RDA was used to assess the root morphological characteristics among the first three orders of *P. yunnanensis* roots under different months. The findings revealed that the first two axes of the RDA represented roughly 65% of the overall variance among all seedling orders (Figure 6A–C and Figure S1A–C). The first and second ordination axes correspondingly indicated the variation in individual biomass, root morphological traits, and NSC concentration. Under the same seedling order, the SRL, SRA, SS, ST, and NSC concentrations in plots had a decent extent of separation (Figure 6A–C and Figure S1A–C). The relationship among SRL, SRA, SS, ST, and NSC concentrations in three seedling orders showed the same results (Figure 6D and Figure S1D).

**Figure 7:**
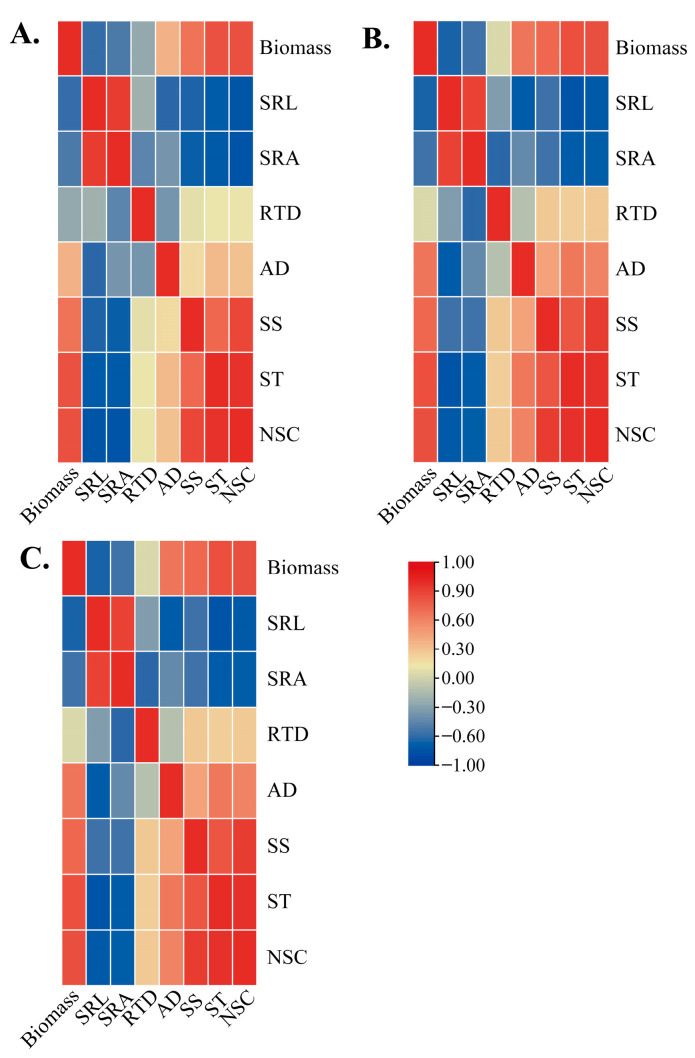
Relationships among biomass, root morphological traits, soluble sugar, and starch concentration in different *P. yunnanensis* seedling orders during the whole experiment period. (**A**) First-order seedlings; (**B**) second-order seedlings; (**C**) and third-order seedlings. SRL: specific root length; SRA: specific root surface area; RTD: root tissue density; AD: average diameter; SS: soluble sugar; ST: starch; NSC: non-structural carbohydrates.

The individual seedling biomass and root morphology of the three orders of roots of *P. yunnanensis* seedlings on different sampling dates were analyzed by using the Pearson correlation coefficient. The results showed that among different seedling orders, the individual biomass of *P. yunnanensis* seedlings was positively correlated with SS, ST, and NSC. The SRL and SRA had an adverse association with SS, ST, and NSC concentration. In contrast, the RTD in three seedling orders had no significant correlation with SS, ST, and NSC (Figure 7A–C). The AD in second-order and third-order seedlings was positively correlated with SS, ST, and NSC, whereas the AD of first-order seedlings was only significantly correlated with ST (Figure 7A).

## 4. Discussion

### 4.1. Relationship Between Biomass Allocation and Root Morphology of Different Seedling Orders

In this study, the biomass of *P. yunnanensis* in needles was significantly higher than that in roots and stems. Recent research has indicated that the biomass allocation is influenced by both the development of the plant and its leaf characteristics. Furthermore, the distribution of biomass to different organs (such as leaves, stems, and roots) is frequently utilized to gain a more comprehensive understanding of a plant’s survival tactics [77,78]. The findings of these research studies correspond with this study’s outcomes and offer significant insights into the physiological and ecological processes of the plant population. He et al. [79] concluded that biomass allocation to leaves and roots increased with the increasing height and height–diameter ratio. In this study, the lower-order seedlings with a higher height and height–diameter ratio showed similar variation patterns.

The SRL, SRA, and other root morphological traits are capable of effectively influencing their water uptake capability [79]. Previous studies have demonstrated that plant roots exhibit greater water absorption efficiency with longer root lengths and smaller root diameters [80,81,82]. Ostonen et al. [41] proposed the following two strategies describing how plants boosted the root system’s absorbing capability to adapt to arid environments: (1) the acquisition strategy, which was to maximize root production while preserving a higher intake surface area; and (2) the conservative strategy, which was to maximize efficiency by altering the morphological traits of the root system. In this research, with the rise of seedling orders, the SRL and SRA increased significantly, which demonstrated that the higher-order seedlings with thinner roots changed their root morphological traits progressively to enhance their absorption capacity. These results confirmed the first hypothesis and were consistent with previous studies: the efficiency of water or nutrient absorption in roots increases when the SRL or SRA increases and the AD decreases [80,81]. Generally, the findings of this investigation highlighted the adaptive strategies of *P. yunnanensis* roots in three different seedling orders.

### 4.2. Effects of Different Seedling Orders on NSC

The shift in NSC with the seasons suggested that the roots and stems were the main storage site of NSC in *P. yunnanensis*, consistent with that of other woody plants [83], supporting that NSC provides essential nutrients for plants [84]. In this study, the SS-to-ST ratio of roots and stems generally increased with the increasing seedling orders. This result showed that plants might change the SS-to-ST ratio to balance the trade-off between needles and roots and improve water transport efficiency, which was consistent with previous studies [85]. This study showed that the total NSC concentration of lower-order seedlings was higher than that of higher-order seedlings, which was similar to the results in *Pinus tabuliformis* Carr. [86] that implied that thick roots transport nutrients to support the entire root, whereas thin roots (absorptive roots) might obtain more significant carbon investment because the primary function was to absorb water and nutrients. These results, along with those of the present study, revealed the interaction of different seedling orders, SS concentration, SS-to-ST, and NSC concentration of *P. yunnanensis* seedlings.

### 4.3. Linking the Root Morphological Traits to the NSC Level

The root system serves as the primary organ in charge of intaking water and was the initial indicator of various stresses [22]. Although Yang et al. [23] have pointed out that thick roots increase NSC accumulation, the premise of this conclusion is that roots build up their unique response strategies under drought conditions. The relationship between root morphological traits and NSC allocation is still open to discussion. In this study, with the increase in the seedling orders, the biomass of *P. yunnanensis* seedlings declined, and the root morphological traits changed to facilitate the intake of water and nutrients (Figure 2A–C and Figure 3A–D).

NSC played a crucial role in root physiological regulation and could promote root growth [87]. In the present study, SRL and SRA of all seedling orders were significantly negatively correlated with the SS, ST, and NSC concentrations. AD was positively correlated with the SS, ST, and NSC concentrations in general (Figure 6A–D and Figure 7A–C). The results confirmed that compared with thicker roots, when the roots were narrower, the thinner ones had more negligible NSC accumulations to sustain normal physiological activities [88]. The finding in this study (Figure 5A–C) supported the anterior result: with a thicker root diameter, plants could easily distribute more carbon to the roots to obtain a more remarkable absorptive ability. Consequently, our findings indicated that the difference in NSC resulting from distinct seedling orders may be associated with variations in root morphological traits.

### 4.4. Seasonal Variation Patterns of NSC Caused by Seasonal Variation of Root Morphological Traits

The ST and SS concentrations in coniferous needles vary in seasons [77,78]. The seasonal variation of the soluble sugar and starch concentration in *P. yunnanensis* is apparent, and the starch and NSC concentration in needles are significant indicators of physiological strategies for coping with seasonal variation [70]. The SS, ST, and NSC concentrations of *P. yunnanensis* in needles were essential indicators of phenotypic plasticity, and the degree of variation in the concentration reflected the degree of the *P. yunnanensis* seedlings’ responses to seasonal variation [70]. The SS concentration in needles of three orders in this study had accumulated, causing the highest needle SS concentration in *P. yunnanensis* in March. During the growing seasons, the SS concentration in needles and ST concentration hydrolyzed into SS concentration were utilized for growth and development. Therefore, the needle SS concentration tended to decrease to the minimum levels in June or September, consistent with those of the previous results [89].

Large NSC reserves in stems could be utilized seasonally, which, as a survival strategy, ensured the resprouting of woody plants after drought, fire, or other disasters [90,91]. NSC storages in roots were crucial for basal resprouting in ecosystems [92,93]. The findings of this study, which were consistent with the observations of the previous study [94], denoted that the ST concentration in roots generally reached the minimum level before June. The seasonal variation patterns of the ST concentration showed a synchrony with that of the SS concentration, demonstrating that root starch in *P. yunnanensis* seedlings serves as a rapid and easily accessible intra-seasonal reserve during growth. The SS concentration in the roots of all the seedling orders declined between September and December, while the ST accumulation of roots in December reached its maximum. The consumption of SS and the preservation of ST might be the survival strategy of *P. yunnanensis* to deal with the drought in the winter season; this finding, in accordance with the second hypothesis, was previously confirmed [22].

In the previous discussions of this present study, the results confirmed the relationship between root morphology variation and the variation of SS, ST, and NSC. Based on this conclusion, it can be assumed that the seasonal variation of NSC is related to the seasonal variation of root morphology, which supported the third hypothesis. Though seasonal variation in total NSC concentration of different seedling orders in different organs generally showed consistency, as for the slight difference in root ST concentration between third-order and the first two-order seedlings, it probably was caused by seasonal variation of root morphology among third-order seedlings.

This study still exists with the limitation of not further exploring the relationship between fine roots and NSC concentration. Therefore, the specific mode of interaction between the fine root system and NSC concentration of *P. yunnanensis* seedlings needs to be further investigated in future research.

## 5. Conclusions

This study investigated the interactive response of root morphological traits and NSC concentration of *P. yunnanensis* seedlings to seedling orders. The *P. yunnanensis* seedlings of different orders exhibited consistent adaptive strategies to seasonal changes in environmental factors, particularly regarding root morphological traits and NSC allocation.

The SRL and SRA of three seedling orders presented significant similarity in responses to seasonal variation, demonstrating a comparable reflection of root water and nutrient absorption. The SRL and SRA values for all seedling orders reached their maximum in March or June, assuming that *P. yunnanensis* altered their root morphology to enhance root absorptive capacity, thereby supporting seedling growth. The morphological characteristics of the roots led to a progressive accumulation of NSC concentration in various organs starting in March. These NSC reserves are partially utilized by *P. yunnanensis* seedlings throughout the growing seasons to adapt to seasonal variation. The total NSC accumulation in roots, stems, and needles across three seedling orders all peaked in December; it would contribute to the growth potential of *P. yunnanensis* seedlings in the next year. The variation patterns of SS and ST concentration in roots across three orders might reveal the adaptive strategy of *P. yunnanensis*. Correspondingly, a significant correlation occurred between root morphology and NSC concentration in principal component analysis and Pearson correlation analysis, and these conclusions revealed that root morphological traits, SS, ST, and NSC concentration were prominent indicators of physiological strategies to cope with seasonal variation.

The results provide information on the seasonal variation of root morphological traits and NSC concentration, which improves our comprehension of the relationship between root morphology and NSC concentration in different orders and provides information useful for the breeding of *P. yunnanensis*.

## Figures and Tables

**Table 1:** Carbon, nitrogen, phosphorus, and potassium contents of the soil in which the seedlings were growing.

Seedling Order	C (g/kg)	N (g/kg)	P (g/kg)	K (g/kg)
I	1.683 ± 0.240 a	2.725 ± 0.099 a	2.079 ± 0.265 a	8.050 ± 0.029 b
II	1.202 ± 0.524 a	1.963 ± 0.303 b	1.832 ± 0.242 a	8.067 ± 0.060 b
III	0.842 ± 0.318 a	3.037 ± 0.105 a	1.850 ± 0.243 a	8.800 ± 0.100 a

I, the first-order seedlings; II, the second-order seedlings; III, the third-order seedlings. The different lowercase letters indicate significant differences among different seedling orders (*p* < 0.05).

**Table 2:** Results of two-way (Time intervals × Seedling orders) ANOVA of *Pinus yunnanensis* roots biomass and root morphological traits.

Source of Variation	df	*p* Values (<0.05)				
		Biomass	SRL	SRA	RTD	AD
Time intervals	3	**0.00**	**0.00**	**0.00**	**0.00**	**0.00**
Seedling orders	2	**0.00**	**0.00**	**0.00**	**0.00**	**0.02**
T × S	6	**0.00**	**0.00**	**0.00**	**0.00**	0.33

T, time intervals; S, seedling orders. *P. yunnanensis*; *Pinus yunnanensis*. Values in bold indicate significant effects.

**Table 3:** Results of two-way (Time intervals × Seedling orders) ANOVA of *P. yunnanensis* soluble sugar (SS), starch (ST), and total NSC.

Source of Variation	df	*p* Values (<0.05)		
		SS	ST	NSC
Time intervals	3	**0.00**	**0.00**	**0.00**
Seedling orders	2	**0.02**	**0.00**	**0.00**
T × S	6	**0.00**	**0.01**	0.70

T, time intervals; S, seedling orders. Values in bold indicate significant effects.

**Table 4:** The SS-ST ratio across different *P. yunnanensis* seedling orders under different time intervals.

Seedling Orders	Time Intervals	SS-ST Ratio in Roots	SS-ST Ratio in Stems
First-order	03/23	0.220	1.775
	06/23	1.726	1.349
	09/23	1.838	1.425
	12/23	0.370	0.861
Second-order	03/23	0.250	1.809
	06/23	0.713	2.280
	09/23	1.178	1.340
	12/23	0.576	0.718
Third-order	03/23	0.305	1.999
	06/23	0.856	1.196
	09/23	2.629	1.296
	12/23	0.511	2.427

## Data Availability

The original contributions presented in the study are included in the article/Supplementary Material, further inquiries can be directed to the corresponding author.

## Supplementary Materials

The following supporting information can be downloaded at: https://www.mdpi.com/article/10.3390/plants14050825/s1, Supplementary Figure S1: Redundancy analysis of biomass of different organs, root morphological traits, and NSC in different seedling orders among different time intervals.
