# Supplementary material for: Seasonal Variation in Root Morphological Traits and Non-Structural Carbohydrates of Pinus yunnanensis Seedlings Across Different Seedling Orders
Source: Plants (Basel). 2025 Mar 6;14(5):825. doi: 10.3390/plants14050825 (PMC11902860; doi:10.3390/plants14050825)
Supplement: Supplementary file 1 [file plants-14-00825-s001.zip › plants-3337039-supplementary.pdf]

Supplemental information for **Seasonal Variation in Root Morphological Traits and Non-structural Carbohydrates of *Pinus yunnanensis* Franch. Seedlings Across Different Seedling Orders**

ZiXing Pan 1,2, ZhuangYue Lu 1,2, SunLing Li 3, JianZhen Liao 1,2, Chi Yu Zhou 1,2, Lin Chen 1,2 , Shi Chen 1,2 , NianHui Cai 1,2, DeXin Wang 1,2* and YuLan Xu 1,2*

1 Key Laboratory of National Forestry and Grassland Administration on Biodiversity Conservation in Southwest China, State Forestry Administration, Southwest Forestry University, Kunming, 650224, Yunnan, China; pzixingg@163.com(Z.P); Luzhuangyue@swfu.edu.cn(Z.L); 18313173051@163.com(J.L); zhouchiyu@swfu.edu.cn(C.Z); linchen@swfu.edu.cn(L.C); chenshi@swfu.edu.cn(S.C); cainianhui@swfu.edu.cn(N.C); woshixin2007@126.com(D.W); xuyulan@swfu.edu.cn(Y.X)

2 Key Laboratory of Forest Resources Conservation and Utilization in the Southwest Mountains of China, Ministry of Education, Kunming, 650224, Yunnan, China;

3 Yunnan Academy of Forestry and Grassland, Kunming, 650224, Yunnan, China; lisunling@yafg.ac.cn(S.L)

* Correspondence: DeXin Wang (Email: woshixin2007@126.com(D.W)); YuLan Xu (Email: xuyulan@swfu.edu.cn))

The following supporting information is available for this article:

Supplementary Figure S1. Redundancy analysis of biomass of different organs, root morphological traits and non-structural carbohydrates (NSC) in different *P. yunnanensis* seedling orders among different sampling dates.

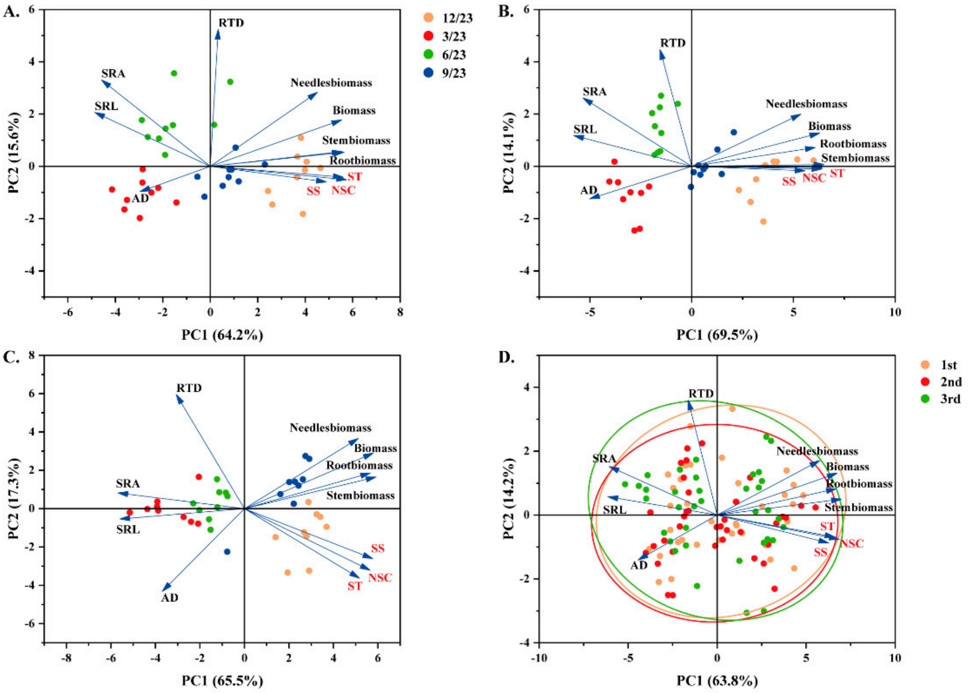

Figure S1. Redundancy analysis of biomass of different organs, root morphological traits and non-structural carbohydrates (NSC) in different *P. yunnanensis* seedling orders among different time intervals. (A) The 1st-order seedlings; (B) the 2nd-order seedlings; (C) the 3rd-order seedlings; (D) redundancy analysis of three seedling orders during the whole experiment period. SRL: specific root length; SRA: specific root surface area; RTD: root tissue density; AD: average diameter; SS: soluble sugar; ST: starch; NSC: non-structural carbohydrates.
